# Utilization of CDX2 Expression in Diagnosing Pancreatic Ductal Adenocarcinoma and Predicting Prognosis

**DOI:** 10.1371/journal.pone.0086853

**Published:** 2014-01-29

**Authors:** Wenbin Xiao, Hong Hong, Amad Awadallah, Lan Zhou, Wei Xin

**Affiliations:** 1 Department of Pathology, University Hospitals Case Medical Center, Cleveland, Ohio, United States of America; 2 Department of Pathology, Case Western Reserve University, Cleveland, Ohio, United States of America; University of Nebraska Medical Center, United States of America

## Abstract

CDX2, a master transcriptional regulator of intestinal cell differentiation and survival, has been used as a marker to indicate colorectal lineage in adenocarcinomas of unknown origin. Pancreatic ductal adenocarcinoma (PDAC) is one of the most common causes for adenocarcinomas of unknown origin, but CDX2 expression in pancreatic disease remains unclear. In this study, we systemically and extensively investigated the expression and role of CDX2 in PDAC. We reported that CDX2 expression is weak and heterogeneous is all normal pancreas and chronic pancreatitis. It is largely expressed in epithelial-lining cells of pancreatic ducts including main ducts, inter-lobular ducts, intra-lobular ducts, intercalated ducts and centroacinar cells, but not in acinar cells or islet cells. CDX2 expression is down regulated during the transformation process from PanIN to PDAC. Only one third of PDACs retain some degree of CDX2 expression, and this group of PDACs have reduced median survival time compared to that of CDX2 negative group (308 days vs. 586 days, p = 0.0065). Metastatic PDACs remain similar expression pattern to that of the primary sites. Our study clearly demonstrates CDX2 expression in pancreatic diseases including PDAC, which is practically important when CDX2 is used to establish the primary sites of adenocarcinomas of unknown origin. In addition, our study also provides CDX2 as a prognostic marker for PDAC and implicates an important role of CDX2 in the development of normal pancreas and PDAC.

## Introduction

CDX2 is a homeobox domain-containing transcription factor that plays an important role in intestinal development by regulating the proliferation and differentiation of intestinal cells [1], [2], [3]. CDX2 is expressed within nuclei of epithelial cells of the intestine from the proximal duodenum to the distal rectum, but very limited expression in esophagus and stomach, therefore CDX2 expression is indicative of intestinal differentiation [1]. Intestinal metaplasia of the gastric mucosa was demonstrated in transgenic mice engineered to express this transcription factor in gastric epithelial cells, including the development of goblet cells expressing acidic-type mucin, enterocyte-like cells expressing alkaline phosphatase and enteroendocrine-type cells [4], [5]. In line with this, in humans, intestinal metaplasia of the stomach and esophagus is consistently accompanied by CDX2 expression [6], [7], [8], [9], [10].

By immunohistochemistry, CDX2 is expressed uniformly in the majority of the colorectal and duodenal adenocarcinoma but is largely negative in the carcinomas of the genitourinary and gynecologic tracts, breast, lung, and head and neck [9], [11], [12], [13]. CDX2 has thus been widely applied to help establish gastrointestinal (GI) origin–and intestinal differentiation in particular–in metastatic tumors. However, strong uniform expression of CDX2 was noted in certain types of tumor outside of GI tract such as mucinous ovarian carcinomas and adenocarcinomas primary to the urinary bladder [11], [13]. Moreover, small portions of gastric and esophageal adenocarcinoma heterogeneously express CDX2 [13]. Therefore, CDX2 expression is not completely specific for carcinoma with GI origin.

With regard to CDX2 expression in normal pancreas and pancreatic ductal adenocarcinoma (PDAC), data extracted from several previous studies are very inconsistent. Werling et al and Chu et al both reported heterogeneous CDX2 expression in 32% (7 of 22 cases) and 22% (10 of 46 cases) of PDAC [13], [14], which has been challenged by others showing no CDX2 expression in PDAC [11], [15]. Regarding CDX2 expression in normal pancreas, Moskaluk et al demonstrated focal and moderate to strong CDX2 expression in ductal lining cells and centroacinar cells but not acinar cells [12]. Kaimaktchiev et al also noticed light staining of epithelial cells lining small ducts in the pancreas, but no nuclear staining in cells lining the main pancreatic duct [11]. However, the main pancreatic duct showed in their study clearly had the morphology of PanIN-1, which undermines their conclusion.

Since PDAC is one of most common origin for adenocarcinoma of unknown primary and CDX2 has been widely used to establish the primary site, it is of importance to clarify CDX2 expression in PDAC. Therefore, we set up to investigate the CDX2 expression in PDAC as well as it precursor lesions–PanIN, with comparison to normal pancreas and chronic pancreatitis. We also compared CDX2 expression in metastatic PDAC to the primary ones. We further explored the prognostic value of CDX2 expression in PDAC.

## Materials and Methods

### Sample Collection

Hematoxylin- and eosin-stained sections retrieved from the files of the Department of Pathology; University Hospitals Case Medical Center, were reviewed. We selected 61 cases of primary pancreatic ductal adenocarcinoma, 21 of normal pancreatic tissue, and 25 of chronic pancreatitis. The normal pancreatic tissue was from patients with non-pancreatic neoplastic disease, most of which were Whipple resections of chronic pancreatitis. Cases with pancreatic intraepithelial neoplasia (PanINs) were included as well: 13 PanIN 1, 12 PanIN 2 and 22 PanIN 3. All PanIN 1 and 2 cases are not associated with PDACs, while all PanIN3 cases are associated with PDACs. Only 1 case of the total 61 PDACs was intestinal type. Metastatic PDAC in peripancreatic lymph nodes was available in 11 cases. In addition, 14 patients with metastatic PDAC in liver and lung were selected. All these patients had undergone surgical resection between 2001 and 2005, with no neoadjuvant chemotherapy or radiotherapy. All specimens analyzed were formalin-fixed and paraffin-embedded tissue sections. The study was approved by the Institutional Research Board (IRB) of the University Hospitals Case Medical Center, and the written consent was waived based on that the study was on the discarded tissues and charts review only.

### Immunohistochemical (IHC) Staining

Expression levels of CDX2 were examined by immunohistochemistry (IHC) on all cases included in this study. The IHC was performed by the Immunohistochemistry Laboratory of University Hospitals Case Medical Center. Briefly, unstained 4 µm-sections of tissue microarrays were prepared from paraffin blocks and baked for 30 minutes at 60°C in a Boekel Lab oven. The slides were then processed using a BenchMark XT (Ventana) automated immunostainer. The slides were deparaffinized, antigen retrieved with standard Cell Conditioning 1 (Ventana Medical Systems, AZ, USA), a tris-based buffer pH 8.3 solution for 30 minutes at 100°C, then incubated at 37°C with the primary antibody CDX2 rabbit monoclonal EPR2764Y (Cell Marque Corp, CA., USA.) for 32 minutes and subsequently counterstained. Nuclear immunoreactivity was considered as a positive expression. Immunoreactivity was scored by two investigators based on the percentage of positive epithelium cells (percentage: 0: <1%, 1+: 1–25%, 2+: 25–50%, 3+: 50%–75%, 4+: 75%–100%). Score 0 was considered as negative. The intensity was scored as weak, moderate and strong. We define the CDX2 intensity of colorectal cancer as strong.

### Statistical Analysis

Comparison of the CDX2 expression rates among different groups was done using the Fisher's exact test (two-tailed).

### Ethic Statement

The research protocol was approved by the Institution Review Board at University Hospital at Case Medical Center, and the written consent was waived based on that the study was on the discarded tissues and charts review only.

## Results

### CDX2 Expression in Normal Pancreas and Chronic Pancreatitis

**Figure 1:**
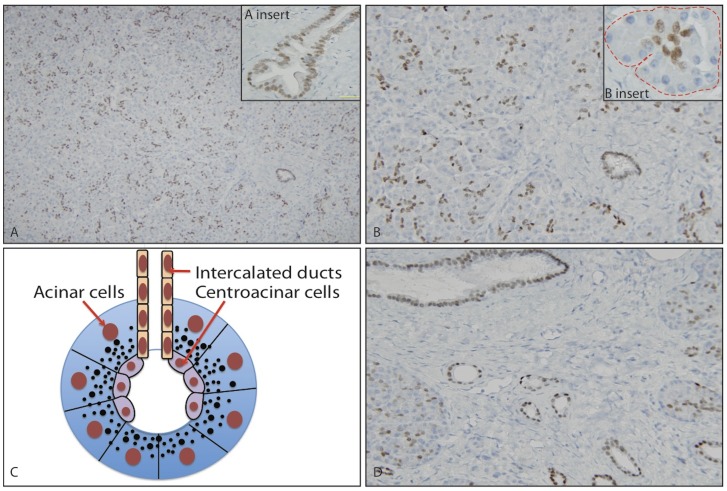
CDX2 expression in normal pancreas and chronic pancreatitis. A. Moderate and heterogeneous CDX2 expression in normal pancreas (×20). Insert shows CDX2 expression in normal ducts. B. CDX2 is expressed in centroacinar cells, intercalated ducts, intralobular ducts and interlobular ducts, but not in acinar cells (×40). Insert shows CDX2 expression in centroacinar cells but not in acinar cells. C. Diagram illustrating the relationship between acinar cells and centroacinar cells. D. CDX2 expression in chronic pancreatitis (×20).

CDX2 expression levels were evaluated by immunohistochemical analysis in normal pancreas and chronic pancreatitis resected with no history or evidence of PDACs. As shown in Figure 1, all normal pancreas examined had scattered heterogeneous and moderate positive nuclear signals, as contrast to the homogenous and strong nuclear staining pattern in colorectal adenocarcinoma. In detail, acinar cells and islets were exclusively negative for CDX2 staining, but intercalated ducts, intralobular and interlobular ducts were relatively homogeneously moderate positive. Centroacinar cells, an inherent component of intercalated ducts that extends to the acinar, were also positive for nuclear CDX2 staining. Interestingly, chronic pancreatitis had similar CDX2 staining pattern to normal pancreas. Intercalated ducts, intralobular ducts, and interlobular ducts were positive for CDX2 with moderate intensity, but acinar cells and fibrous stromal cells were negative.

### CDX2 Expression in PDAC and its Precursor Lesions

**Figure 2:**
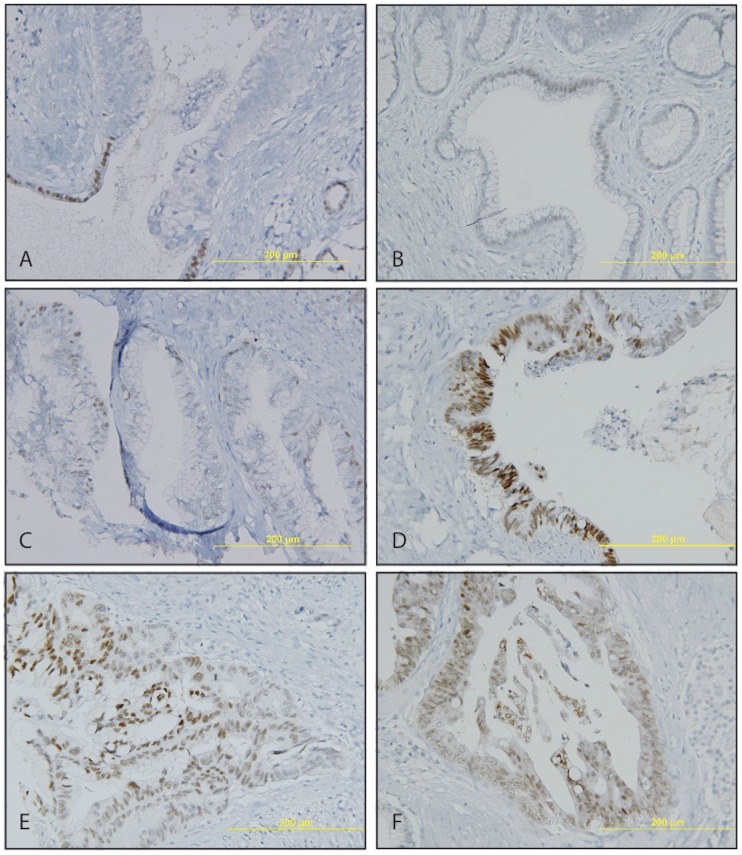
CDX2 expression in PanINs. A. CDX2 expression is lost during the transition from normal duct to PanIN1/2 (×20). B–F. A wide spectrum of CDX2 expression in PanIN (×20). B. PanIN1. C–D, PanIN2. E–F, PanIN3.

PanINs have recently been proposed as noninvasive precursor lesions of PDACs. PanINs are believed to progress from PanIN1 to 3 [16]. We therefore examined CDX2 expressions in PanINs. Nuclear CDX2 staining was observed from PanIN 1 to 3 (Figure 2). The weak and heterogeneous staining pattern, in PanIN was similar to that in PDAC. About 60–70% of PanIN1 (61.5%) and PanIN2 (75%) were positive for CDX2 (Table 1), significantly lower than that of normal pancreas. Moreover, only 31.8% of PanIN 3 was positive for CDX2.

**Table 1:** CDX2 expression in normal pancreas, chronic pancreatitis, PanIN and PDAC.

	Normal ducts	Acinar	Chronic pancreatitis	PanIN1	PanIN2	PanIN3	PDAC
CDX2+ (N)	21	0	25	8	9	7	22
CDX2− (N)	0	21	0	5	3	15	39
Total	21	21	25	13	12	22	61
CDX2+ (%)	100	0	100	61.5*	75*	31.8*	36.1* ^,^ **

* p<0.01 vs. normal ducts,

** p<0.05 vs. PanIN2.

**Figure 3:**
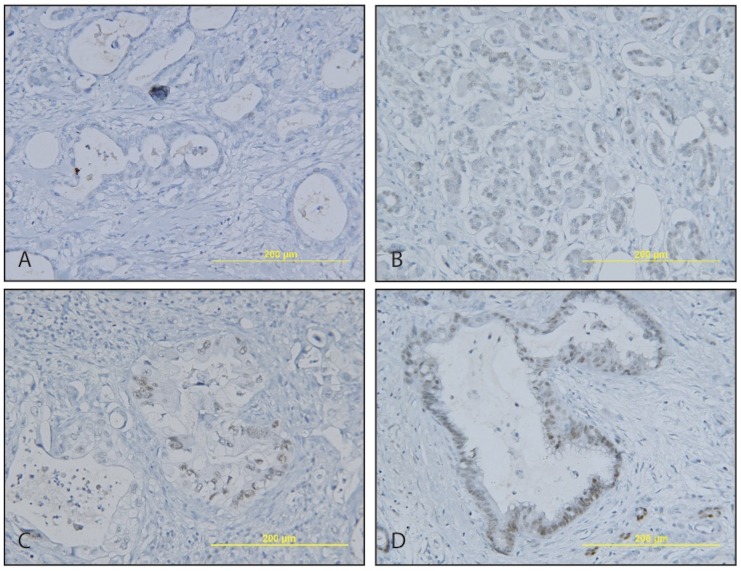
CDX2 expression in PDAC. CDX2 is largely negative (A) in PDAC, but can be weakly (B) and moderately positive (C and D) (×20).

We next accessed CDX2 expression in PDACs. As expected, the majority (63.9%) of PDACs had no detectable CDX2 expression (Figure 3 and Table 1). However, 36.1% of PDAC weakly expressed CDX2 in the nuclei of malignant cells. The staining pattern was weak, scattered and heterogeneous, in contrast to moderate and homogeneous positivity in normal intercalated ductal cells. These results indicate that CDX2 expression is lost in about two thirds of PDAC and is largely reduced in remaining one third of PDAC.

### CDX2 Expression in Metastatic PDAC

**Figure 4:**
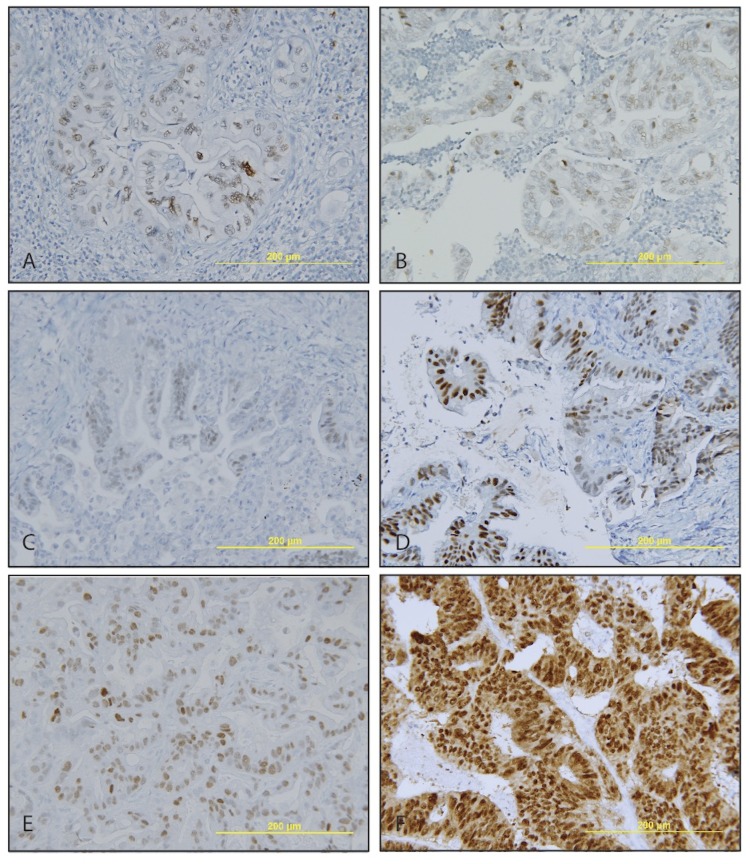
Weak to moderate and heterogeneous CDX2 expression in metastatic PDAC. A–B, Metastatic PDAC in peripancreatic lymph nodes. C–D, Metastatic PDAC in lung. E, Metastatic PDAC in liver. F, Metastatic colonic adenocarcinoma in liver served as a control.

We further investigated CDX2 expression in metastatic PDAC since one third of primary PDAC retains weak and heterogeneous CDX2 expression. To this end, we had 11 cases available with peripancreatic lymph node metastasis, of which 4 (36.4%) were positive for CDX2 expression (Figure 4A-B and Table 2). The metastatic carcinoma in lymph nodes show the identical CDX2 expression patterns as those of primary PDACs, with a 100% concordance on CDX2 expression between primary and metastatic PDAC, demonstrating that PDAC retains CDX2 expression after lymph node metastasis.

**Table 2:** Comparison of CDX2 expression between primary PDAC and metastatic PDAC.

CDX2 expression	Primary PDAC N(%)	Metastatic PDAC in LN N(%)	Metastatic PDAC in non-LN N(%)
0	39 (63.9)	7 (63.6)	10 (71.4)
1+	8 (13.1)	0 (0)	3 (14.4)
2+	9 (14.8)	1 (9.1)	1 (7.1)
3+	4 (6.6)	3 (27.3)	1 (7.1)
4+	1 (1.6)	0 (0)	0

LN, lymph node.

We also examined CDX2 expression in resection specimens from 14 patients presented with metastatic PDAC in liver or lung (Figure 4C-E and Table 2). Only 4 (28.6%) out of 14 showed weak and heterogeneous CDX2 expression. Not surprisingly, the metastatic carcinomas have the same CDX2 expression pattern as those of primaries. Taken together, PDACs retain weak and heterogeneous CDX2 expression pattern after lymph node and/or distal organ metastasis their primary PDACs. No metastatic PDACs regain CDX2 expression if the primary carcinomas are negative for CDX2.

### CDX2 Expression Contributes to the Inferior Survival in Patients with PDAC

**Figure 5:**
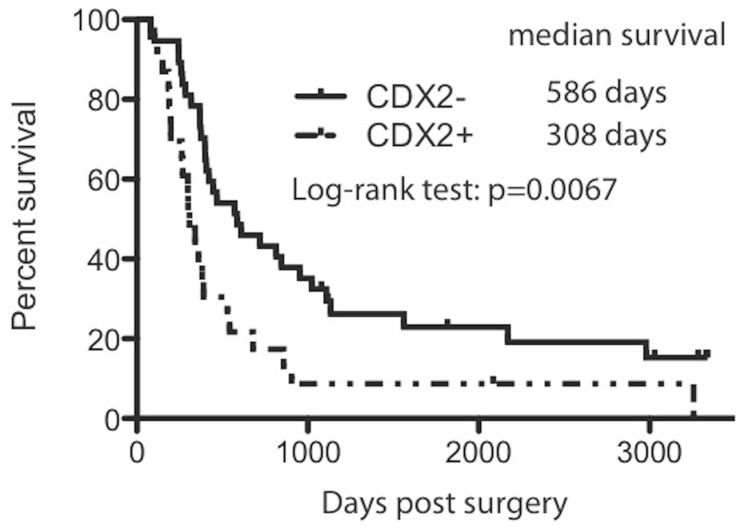
CDX2 expression is associated with inferior survival in PDAC.

We next asked whether CDX2 expression correlates with the clinical outcome in the group of 61 patients who had surgery done between 2001 and 2005. We observed that the expression of CDX2 is associated with shorter survival (Figure 5). Patients (n  = 22) whose tumor showed CDX2 immunoreactivity had a shorter median survival time of 308 days, whereas patients (n = 39) whose tumor did not show CDX2 immunoreactivity had a mean survival time of 586 days (Log-rank test, P = 0.0067). Furthermore, we did not find any other factors, such as age, gender, tumor size, and differentiation, which are clearly associated with prognosis (Table 3).

**Table 3:** Demographic data of patients with PDAC.

	CDX2− (n = 39)	CDX2+ (n = 22)
Gender		
Male	20	12
Female	19	10
Age (years)		
Range	36∼80	30∼82
Mean±SD (median)	64.1±11.2 (68)	63.7±12.3 (67)
Tumor location		
Head	39	17
Tail	0	5
Tumor size (cm)		
Range	1.1∼8	1∼6.5
Mean±SD (median)	3.5±1.9 (3)	3.9±2.0 (3.8)
Tumor differentiation		
Moderate	31	13
Poor	8	9
Resection margin		
Negative	23	13
Positive	16	9
Lymph node metastasis (n)		
0	8	3
1	4	6
2	4	3
≥3	23	10

## Discussion

PDAC is the fourth leading cause of cancer death and is usually presented with locally advanced or metastatic disease [16]. PDAC, in addition to lung adenocarcinoma, is one of the most common causes for metastatic adenocarcinomas of unknown primary. A panel of immunohistochemical markers including TTF-1, CDX2, CK7 and CK20, has been used to determine the tissue origin of these metastatic adenocarcinomas. In this context, it’s been demonstrated that a multiple-marker panel of immunohistochemical staining consisting of TTF-1−/CDX2−/CK7+/CEA+/MUC5A+ has a 98% specificity for pancreaticobilirary carcinoma, but the sensitivity is as low as 28.4% [17]. The low sensitivity suggests that the majority of pancreaticobilirary carcinoma doesn’t follow the above staining pattern. One good example was shown in this study that CDX2 is in fact expressed in about one third of PDAC, against the traditional view of deadly negative CDX2 expression in PDAC. In this regard, an in-depth understanding of CDX2 expression in PDAC is of critical importance for surgical pathologists to appropriately establish PDAC as a differential diagnosis.

Although CDX2 expression in PDAC has been briefly mentioned in a few studies, the results were somehow contradictory, for example, the percentage of CDX2 positive PDAC ranged from 0–50% depending on individual study [11], [13], [14], [15]. One possibility among many others is that in most of the previous studies CDX2 expression in pancreas (including PDAC) was compared to that in colon (including colon cancer) [11], [12], [13]. Therefore, weak and scattered positivity might be overlooked if strong and homogeneous CDX2 expression in colon cancer is used as a control. In this study we showed CDX2 expression in about one third of PDAC and the expression pattern is weak and heterogeneous in contrast to the strong and homogeneous pattern seen in colon cancer. In normal pancreas, CDX2 expression is heterogeneous, which can be explained by its expression in duct-lining cells including centroacinar cells, but not in acinar cells. Interestingly, chronic pancreatitis has similar CDX2 expression pattern to normal pancreas. As PDAC is originated from ductal cells, our results implicate that CDX2 protein expression in PDAC is largely downregulated compared to normal ductal epithelium. Several studies also showed CDX2 mRNA expression is significantly lower in PDAC than in normal pancreas and chronic pancreatitis (data available at Oncomine database www.oncomine.org) [18], [19]. Along the same lines, it has been shown that CDX2 expression is downregulated in several types of PDAC cell lines at the levels of mRNA and proteins [20].

The role of CDX2 in pancreas is unknown. Mice heterozygous for CDX2 do not show pancreatic defects [3], [21]. However, it remains possible that low levels of CDX2 might be sufficient for appropriate development of the pancreas. As conventional knockout mice for CDX2 are embryonic lethal [2], [21], conditional CDX2 knockout mice specific in pancreatic cell lineage will probably address this puzzle. We showed loss of or reduced CDX2 expression in PanIN1-3, and PDAC in a stepwise manner. As PanIN has widely been accepted as noninvasive precursor lesions of PDAC [16], our data suggest that loss of CDX2 expression is an early event for tumorigenesis in pancreas. In this regard, CDX2 might function as a tumor suppressor in pancreas to suppress the development and/or progression of PanIN. We showed loss of or reduced CDX2 expression from normal pancreas to PanIN, and to PDAC in a stepwise manner. As PanIN has widely been accepted as noninvasive precursor lesions of PDAC [16], our data suggest that CDX2 might function as a tumor suppressor in pancreas. On the other hand, CDX2 could be an oncogene as well. Salari et al recently reported that CDX2 is an amplified oncogene in colorectal cancer that demonstrates a marked dependency on CDX2 levels for continued growth and survival [22]. Mechanistically, CDX2 up regulates Wnt/β-catenin signaling, and is a key oncogenic pathway in colorectal cancer [22]. The potential oncogenic role of CDX2 in PDAC remains to be investigated.

CDX2 expression has been associated with prolonged survival in gastric, ovarian and gallbladder adenocarcinomas [23], [24], [25], [26], [27]. Matsumoto et al reported that CDX2 positive PDAC patients have better outcome than CDX2 negative ones with no median survival data available [28]. In contrast, in our study, the median survival of CDX2 negative PDAC is nearly twice of CDX2 positive ones, clearly demonstrating the association of CDX2 expression and inferior survival in PDAC. There are several notably differences between these two studies. First, the case number in our study is significantly larger. Second, we defined >1% as positive staining compared to >10% used by Matsumoto’s study. Third, the population in two studies is different (western vs. Asian). Interestingly, CDX2 expression also contributes to worse prognosis in leukemia [29]. Therefore, the impact of CDX2 on survival is probably cancer type specific.

In summary, we, in this study, systemically and extensively investigated the expression and role of CDX2 in PDAC with comparison to normal pancreas and chronic pancreatitis. Our findings provide a better understanding on CDX2 in pancreatic diseases and are practically useful. We showed that more than a third of PDACs show a weak and heterogeneous expression pattern of CDX2. The expression pattern is unique and different from that of colorectal lineage which shows strong and uniform expression of CDX2. We suggest that when applying CDX2 as an immunohistochemical marker to determine the source of the primary tumors, the pathologists have to carefully interpret the CDX2 expression pattern instead of arbitrative assign positive and negative expression. Our study also suggests that CDX2 might play important roles in pancreatic development and oncogenic transformation.
